# Trends in Emergency Department Use by Rural and Urban Populations in the United States

**DOI:** 10.1001/jamanetworkopen.2019.1919

**Published:** 2019-04-12

**Authors:** Margaret B. Greenwood-Ericksen, Keith Kocher

**Affiliations:** 1 Department of Emergency Medicine, University of New Mexico, Albuquerque; 2 Department of Emergency Medicine, University of Michigan, Ann Arbor; 3 Institute for Healthcare Policy and Innovation, University of Michigan, Ann Arbor

## Abstract

**Importance:**

Patterns in emergency department (ED) use by rural populations may be an important indicator of the health care needs of individuals in the rural United States and may critically affect rural hospital finances.

**Objective:**

To describe urban and rural differences in ED use over a 12-year period by demographic characteristics, payers, and characteristics of care, including trends in ambulatory care–sensitive conditions and ED safety-net status.

**Design, Setting, and Participants:**

This cross-sectional study of ED visit data from the nationally representative National Hospital Ambulatory Medical Care Survey examined ED visit rates from January 2005 to December 2016. Visits were divided by urban and rural classification and stratified by age, sex, race/ethnicity, and payer. Emergency departments were categorized as urban or rural in accordance with the US Office of Management and Budget classification. Codes from the *International Classification of Diseases, Ninth Revision* (*ICD-9*), were used to extract visits related to ambulatory care–sensitive conditions. Safety-net status was determined by the Centers for Disease Control and Prevention definition. Visit rates were calculated using annual US Census Bureau estimates. National Hospital Ambulatory Medical Care Survey estimates were generated using provided survey weights and served as the numerator, yielding an annual, population-adjusted rate. Data were analyzed from June 2017 to November 2018.

**Main Outcomes and Measures:**

Emergency department visit rates for 2005 and 2016 with 95% confidence intervals, accompanying rate differences (RDs) comparing the 2 years, and annual rate change (RC) with accompanying trend tests using weighted linear regression models.

**Results:**

During the period examined, rural ED visit estimates increased from 16.7 million to 28.4 million, and urban visits increased from 98.6 million to 117.2 million. Rural ED visits increased for non-Hispanic white patients (13.5 million to 22.5 million), Medicaid beneficiaries (4.4 million to 9.7 million), those aged 18 to 64 years (9.6 million to 16.7 million), and patients without insurance (2.7 million to 3.4 million). Rural ED visit rates increased by more than 50%, from 36.5 to 64.5 visits per 100 persons (RD, 28.9; RC, 2.2; 95% CI, 1.2 to 3.3), outpacing urban ED visit rates, which increased from 40.2 to 42.8 visits per 100 persons (RD, 2.6; RC, 0.2; 95% CI, −0.1 to 0.6). By 2016, nearly one-fifth of all ED visits occurred in the rural setting. From 2005 to 2016, rural ED utilization rates increased for non-Hispanic white patients (RD, 26.1; RC, 1.6; 95% CI, 0.4 to 2.8), Medicaid beneficiaries (RD, 56.4; RC, 4.1; 95% CI, 2.1 to 6.1), those aged 18 to 44 years (46.9 to 81.6 visits per 100 persons; RD, 34.7; RC, 2.3; 95% CI, 1.1 to 3.5) as well as those aged 45 to 64 years (27.5 to 53.9 visits per 100 persons; RD, 26.5; RC, 1.6; 95% CI, 0.7 to 2.5), and patients without insurance (44.0 to 66.6 visits per 100 persons per year; RD, 22.6; RC, 2.7; 95% CI, 0.2 to 5.2), with a larger proportion of rural EDs categorized as safety-net status.

**Conclusions and Relevance:**

Rural EDs are experiencing important changes in utilization rates, increasingly serving a larger proportion of traditionally disadvantaged groups and with greater pressure as safety-net hospitals.

## Key Points

**Question:**

How do payer status and patient demographic characteristics differ between urban and rural emergency department (ED) visits?

**Findings:**

In this cross-sectional study of National Hospital Ambulatory Medical Care Survey data, rural ED visit rates increased by more than 50%, from 36.5 to 64.5 per 100 persons, outpacing urban ED visit rates, which increased from 40.2 to 42.8 visits per 100 persons. Rural ED use increased for those aged 18 to 64 years, non-Hispanic white patients, Medicaid beneficiaries, and patients without insurance, with a larger proportion of rural EDs categorized as safety-net EDs.

**Meaning:**

Rural EDs experienced greater growth in ED use simultaneous with increased pressure as safety-net hospitals.

## Teaser

This cross-sectional study of National Hospital Ambulatory Medical Care Survey data describes differences between rural and urban emergency department visits over a 12-year period by demographic characteristics, payers, and characteristics of care.

## Introduction

Recent reports suggest troubling declines in the health of individuals who live in the rural United States, with increases in mortality,^1^ greater rates of chronic disease and high-risk health behaviors,^2^ and widening differences between rural and urban life expectancy.^3,4^ Rural areas are further constrained by physician shortages^5^ and financially stressed hospitals with operating margins often too narrow to invest in upgrades to optimize care delivery.^6^ As a result of these challenges, rural populations may engage with the health care system differently than their urban counterparts. Understanding the health care use of individuals in rural areas may yield insights into addressing these growing health disparities.

Emergency department (ED) use patterns provide a lens into the status of health care delivery in the communities they serve. Emergency departments play a unique and evolving role in the health care system as a site for the unplanned acute care needs of their communities^7^ and as the chief location for admission to the hospital.^8^ Emergency department visits may reflect progression or exacerbations of poorly controlled chronic diseases or potentially signal barriers in access to usual sources of care, such as primary or specialty outpatient settings. However, traditional office-based care settings require significant resource investment and a robust physician pool, which may be lacking in rural communities.^9^ These factors raise the possibility that rural EDs are increasingly serving as a source of care for rural patients in ways that are distinct from their urban counterparts.

To evaluate this hypothesis, we examined changing trends in rural ED visits and assessed for associated drivers. These included patient demographic characteristics and payer status, visit types, and proportion of visits for ambulatory care–sensitive conditions, which can serve as a marker for outpatient care availability. Additionally, we examined the proportion of EDs that met the safety-net classification, as this designation can contribute to the eroding financial solvency of rural hospitals. Our analysis aims to describe use in rural EDs, which, to our knowledge, has never been done previously and has important implications for rural health care delivery.

## Methods

### Data Source and Study Population

To determine the yearly number of ED visits and associated confidence intervals, we analyzed data with provided survey weights from the National Hospital Ambulatory Medical Care Survey (NHAMCS), an annual, national probability sample survey on use and provision of services in hospital-based EDs, from January 2005 to December 2016. We included all visits to hospital-based EDs. We excluded data from 2012, as the urban/rural classification variable was not publicly available. Emergency departments were categorized as urban or rural in accordance with the US Office of Management and Budget classification from the 2010 census for all years.^10^ Because the definition of urban and rural settings can change over time, we also conducted a sensitivity analysis and applied the Office of Management and Budget classification criteria from 2000 and found similar rates.

To determine the reference population on which to generate rates, we used the US Census Bureau estimates of the civilian, noninstitutionalized population, excluding patients in long-term care and incarcerated individuals. These estimates were further divided into urban and rural populations in accordance with the Office of Management and Budget 2010 classifications, then stratified by age, sex, and race/ethnicity. As a result, the Office of Management and Budget definitions for urban and rural were used for NHAMCS data and US Census Bureau data. Therefore, the NHAMCS estimate, generated using provided survey weights, served as the numerator and the US Census estimate, generated by the US Census Bureau, served as the denominator, yielding an annual, population-adjusted rate. This approach is used by NHAMCS in their yearly reports, as detailed in the microdata files,^11^ and was confirmed by personal communication with NHAMCS/National Ambulatory Medical Care Survey statisticians (Don Cherry, oral communication, June 2017).

Finally, we calculated rate differences (RDs) by subtracting the 2005 rate from the 2016 rate as an absolute measure of change. We then calculated the annual rate change (RC) by regressing each year’s rate over time, weighted by the inverse of the variance.

Our analysis was conducted between June 2017 and November 2018. This study was exempted from review by the University of Michigan’s institutional review board, as it uses a publicly available data set that contains no patient identifiers, and informed consent was waived. This study is reported in accordance with the Strengthening the Reporting of Observational Studies in Epidemiology (STROBE) reporting guideline.^12^

### Study Outcomes

Visit rates are reported by age, sex, race/ethnicity, insurance status, triage category, and disposition category (ie, hospital admission or transfer). Ambulatory care–sensitive conditions, a set of diagnoses reflecting the quality and availability of outpatient services, mirror the established definitions of the Agency for Healthcare Research and Quality definitions of prevention quality indicators by validated codes from the *International Classification of Diseases, Ninth Revision* (*ICD-9*).^13^ The conditions included bacterial pneumonia, hypertension, perforated appendix, congestive heart failure, diabetes (uncontrolled or complications), angina, chronic obstructive pulmonary disease, urinary tract infection, and dehydration. Safety-net status was determined by Centers for Disease Control and Prevention criteria, which are based on the proportion of patients without insurance and Medicaid populations served.^14^

### Statistical Analysis

Annual ED visit rates were calculated using the US Census Bureau estimates of the civilian, noninstitutionalized population, which were divided into urban and rural populations in accordance with the Office of Management and Budget 2010 classifications and then further stratified by age, sex, and race/ethnicity. For the purpose of exploring racial/ethnic disparities, we categorized patients as non-Hispanic white, non-Hispanic black, or Hispanic using guidance from the NHAMCS.^15^ The relative standard errors for each categorization for rural and urban are 30% or less, indicating reliable estimates. Annual estimates of persons by insurance type are based on the American Community Survey, starting in 2008^16^; in prior years, insurance status was collected by the Current Population Survey Annual Social and Economic Supplement^17^ without county-level identifiers, which prevents identification of urban and rural populations.

Visit rates are reported with 95% CIs based on standard errors provided by the NHAMCS. We report visit rates for 2005 and 2016 by age, sex, race/ethnicity, payer type, and ambulatory care–sensitive conditions. Additionally, to understand trends over time, we calculated the RD across this 12-year period, an approach previously established in the literature,^18^ along with the annual RC. Rate change was generated by performing weighted linear regression tests of trend to account for the sampling scheme used by the NHAMCS. Weights were the inverse of the variance estimates calculated from the standard errors as described in previous literature.^19^

We additionally reported change in acuity level and disposition category, also with accompanying RD and RC. For 2005 to 2008, the NHAMCS triage category was rated on a 5-point scale, based on the immediacy with which the patient should be seen: (1) immediate, (2) 1 to 14 minutes, (3) 15 to 60 minutes, (4) 1 to 2 hours, and (5) 2 to 24 hours. In 2009, NHAMCS renamed the 5 categories (1) immediate, (2) emergent, (3) urgent, (4) semiurgent, and (5) nonurgent, which we coded as synonymous with the earlier categories.

The number of safety-net EDs was determined by dividing the weighted estimate of urban and rural EDs that met the criteria for safety-net hospitals by the number of urban and rural EDs designated as a service line in the Annual Survey of Hospitals between 2005 and 2016.^20^ The Centers for Disease Control and Prevention defines a safety-net ED as meeting 1 or more of the following criteria: (1) having more than 30% of ED visits with Medicaid as the expected source of payment, (2) having more than 30% of visits with self-pay or no charge as the expected source of payment (considered without insurance), or (3) having a combined Medicaid and uninsured pool greater than 40% of visits.^21^ All analyses were performed in Stata version 14.0 (StataCorp) accounting for the complex survey design. Level of significance was set at *P* = .05, and tests were 2-tailed.

## Results

### Overall Rural and Urban ED Visit Trends

**Figure 1.**
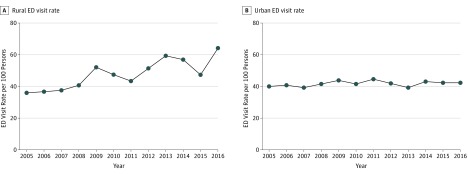
Rural and Urban Emergency Department (ED) Visit Rates from 2005 to 2016

From 2005 to 2016, estimated rural ED visits increased from 16.7 million to 28.4 million and estimated urban visits from 98.6 million to 117.2 million (Table 1 and Table 2), with rural increases in non-Hispanic white patients (13.5 million to 22.5 million), Medicaid beneficiaries (4.4 million to 9.7 million), those aged 18 to 64 years (9.6 million to 16.7 million), and patients without insurance (2.7 million to 3.4 million). Rural ED visit rates increased by more than 50%, from 36.5 to 64.5 per 100 persons (RD, 28.9; RC, 2.2; 95% CI, 1.2 to 3.3) between 2005 and 2016 (Figure 1 and Table 1). This increase outpaced urban ED visit rates, which were generally flat, increasing from 40.2 to 42.8 visits per 100 persons (RD, 2.6; RC 0.2; 95% CI, −0.1 to 0.6) (Figure 1 and Table 2). Detailed information on visit counts, rates, and weighting can be found in the eTable in the Supplement.

**Table 1.** Rural Emergency Department Visits in the United States by Demographic Characteristics, Triage Category, and Disposition in 2005 and 2016

Characteristic	ED Visits, Unweighted No.		Estimated ED Visits, Weighted No. in Millions		Estimated ED Visits per 100 Persons				
	2005	2016	2005	2016	2005, No. (95% CI)	2016, No. (95% CI)	RD	RC (95% CI)	*P* Value
Total ED visits	4047	2759	16.7	28.4	36.5 (17.2 to 55.9)	64.5 (29.3 to 99.7)	28.9	2.2 (1.2 to 3.3)	.001
Visits by age, y									
<18	937	540	3.9	5.8	40.2 (17.7 to 62.8)	61.8 (25.9 to 97.7)	21.6	1.1 (−0.1 to 2.4)	.07
18-44	1538	996	6.5	10.4	46.9 (21.4 to 72.4)	81.6 (37.2 to 126.0)	34.7	2.3 (1.1 to 3.5)	.002
45-64	782	642	3.1	6.3	27.5 (12.8 to 42.3)	53.9 (26.3 to 81.5)	26.5	1.6 (0.7 to 2.5)	.004
≥65	790	581	3.1	5.9	50.0 (23.2 to 76.7)	81.6 (31.8 to 131.4)	31.6	1.1 (−0.7 to 3.0)	.19
Visits by sex									
Male	1874	1293	7.6	13.3	37.1 (17.2 to 56.9)	65.5 (29.9 to 101.2)	26.1	1.6 (0.6 to 2.6)	.006
Female	2173	1466	9.1	15.1	43.8 (19.8 to 67.7)	72.7 (32.8 to 112.6)	28.9	1.7 (0.3 to 3.1)	.02
Visits by race/ethnicity									
White	3464	2298	13.5	22.5	39.2 (17.8 to 60.6)	65.3 (28.5 to 102.1)	26.1	1.6 (0.4 to 2.8)	.01
Black	366	268	2.1	3.4	58.9 (5.6 to 112.1)	95.5 (9.7 to 181.4)	36.6	1.1 (−2.3 to 4.6)	.47
Hispanic	172	161	0.8	2.0	48.6 (6.8 to 90.4)	91.9 (31.2 to 152.7)	43.3	0.9 (−1.9 to 3.7)	.48
Visits by insurance status^a^									
Private insurance	1727	1064	6.8	9.9	30.0 (13.2 to 42.8)	38.3 (17.7 to 58.8)	8.3	0.5 (−0.3 to 1.4)	.21
Medicare	947	722	3.8	6.4	48.7 (22.8 to 74.5)	74.5 (34.0 to 114.9)	25.8	1.3 (−0.6 to 3.2)	.16
Medicaid	1081	969	4.4	9.7	56.2 (24.9 to 87.5)	112.6 (52.6 to 172.5)	56.4	4.1 (2.1 to 6.1)	.001
No insurance	590	330	2.7	3.4	44.0 (14.8 to 73.1)	66.6 (6.5 to 126.7)	22.6	2.7 (0.2 to 5.2)	.04
Visits for ACSCs^b^	429	328	1.3	1.5	3.6 (1.7 to 5.6)	4.5 (2.1 to 6.9)	0.9	0.1 (0 to 0.2)	.13
Visits by disposition category, %^b^									
Hospitalized	357	141	1.6	1.8	9.3 (4.1 to 14.5)	6.3 (1.5 to 11.1)	−3.0	−0.3 (−0.5 to −0.1)	.009
Transferred	149	141	0.6	1.2	3.3 (1.6 to 5.1)	4.2 (1.3 to 7.0)	0.9	0.1 (0 to 0.2)	.05
Visits by triage category^b^									
Immediate	338	7	1.6	0.1	9.6 (1.8 to 17.5)	NA^c^	NA^c^	NA^c^	NA^c^
Emergent	442	140	1.8	1.8	10.9 (2.9 to 18.9)	6.2 (1.2 to 11.4)	−4.7	−0.8 (−1.2 to −0.4)	.002
Urgent	1357	985	5.8	10.5	39.9 (12.0 to 57.9)	36.8 (15.9 to 57.8)	−3.1	−0.6 (−1.3 to 0.2)	.14
Semiurgent	642	755	2.3	8.2	13.7 (4.1 to 23.3)	28.9 (12.5 to 45.3)	15.2	2.2 (0.6 to 3.7)	.01
Nonurgent	439	179	1.8	1.7	10.9 (2.0 to 19.7)	6.1 (1.7 to 10.4)	−4.8	−0.5 (−0.9 to −0.1)	.03
Mean triage category^d^	NA	NA	NA	NA	3.1 (2.8 to 3.4)	3.4 (3.3 to 3.6)	0.3	0.5 (0.2 to 0.7)	.002

Abbreviations: ACSC, ambulatory care–sensitive conditions; ED, emergency department; NA, not applicable; RD, rate difference; RC, annual rate of change.

^a^ These denominators represent 2008 to 2016 because 2008 was the first year the US Census Bureau provided data in a way to allow for identification of urban and rural populations by payer type.

^b^ Denominator is ED visits for each year; proportions are the percentage of total estimated ED visits. Due to *International Statistical Classification of Diseases and Related Health Problems, Tenth Revision *transition occurring in 2015, we reported ACSCs for 2005 to 2015 to avoid invalid comparisons with 2016 data.

^c^ Estimates based on a sample of less than 30 unweighted records are unreliable and are thus not reported.

^d^ Calculated by assigning a value of 1 (most acute) through 5 (least acute) to each of the 5 triage categories and then taking the mean for each year with confidence intervals; *P* value is trend during study for mean triage category.

**Table 2.** Urban Emergency Department Visits in the United States by Demographic Characteristics, Triage Category, and Disposition in 2005 and 2016

Characteristic	ED Visits, Unweighted No.		Estimated ED Visits, Weighted No. in Millions		Estimated ED Visits per 100 Population				
	2005	2016	2005	2016	2005, No. (95% CI)	2016, No. (95% CI)	RD	RC (95% CI)	*P* Value
Total ED visits	29 558	16 708	98.6	117.2	40.2 (33.1 to 47.3)	42.8 (33.9 to 51.6)	2.6	0.2 (−0.1 to 0.6)	.14
Visits by age, y									
<18	7222	3685	25.0	26.4	38.8 (30.1 to 47.5)	41.1 (30.6 to 51.6)	3.1	0.6 (0.02 to 1.2)	.05
18-44	12 458	6570	41.0	45.6	41.3 (34.0 to 48.7)	45.7 (36.1 to 55.3)	4.4	0.5 (0.1 to 0.9)	.01
45-64	5856	3993	19.0	28.1	29.4 (24.5 to 34.3)	39.5 (31.3 to 47.8)	10.1	0.7 (0.4 to 1.0)	.001
≥65	4022	2460	13.6	17.2	42.9 (35.2 to 50.6)	45.7 (35.7 to 55.7)	2.8	0.2 (−0.2 to 0.5)	.33
Visits by sex									
Male	13 690	7599	45.6	52.7	35.6 (29.4 to 41.8)	39.7 (31.5 to 47.9)	4.1	0.4 (0.1 to 0.7)	.03
Female	15 868	9109	53.0	64.5	40.1 (32.9 to 47.3)	46.2 (36.6 to 55.7)	6.1	0.6 (0.4 to 0.9)	.001
Visits by race/ethnicity									
White	16 646	9192	58.3	65.5	35.5 (28.5 to 42.6)	40.2 (30.9 to 49.4)	4.7	0.5 (0.2 to 0.7)	.001
Black	6734	4002	21.2	27.3	64.6 (51.3 to 77.8)	76.8 (57.1 to 96.5)	12.2	1.0 (−0.2 to 2.3)	.09
Hispanic	4927	2717	15.4	20.4	35.3 (25.5 to 45.0)	38.5 (28.7 to 48.3)	3.2	0.7 (0.2 to 1.3)	.01
Visits by insurance status^a^									
Private insurance	11 256	5522	39.2	36.4	22.8 (18.8 to 26.8)	19.9 (15.8 to 24.1)	−2.9	−0.3 (−0.5 to 0.0)	.03
Medicare	4508	2858	15.3	19.5	41.3 (33.3 to 49.3)	46.0 (34.9 to 57.1)	4.7	0.5 (0.0 to 0.9)	.04
Medicaid	8061	6768	24.2	45.3	56.6 (44.4 to 68.8)	88.3 (68.9 to 107.7)	31.7	2.9 (1.6 to 4.4)	.001
No insurance	5331	1714	17.8	12.3	45.7 (36.2 to 55.3)	38.8 (26.5 to 51.0)	−6.9	−0.3 (−1.6 to 1.0)	.62
Visits for ACSCs^b^	3000	1595	7.9	8.3	4.2 (3.4 to 5.0)	3.9 (3.1 to 4.8)	−0.3	0.01 (−0.1 to 0.03)	.12
Visits by disposition category, %^b^									
Hospitalized	3578	1475	12.3	10.9	12.5 (10.1 to 14.9)	9.3 (6.8 to 11.8)	−3.2	−0.4 (−0.7 to −0.1)	.001
Transferred	478	187	1.6	1.2	1.6 (1.2 to 2.0)	1.1 (0.7 to 1.3)	−0.5	−0.1 (−0.1 to 0.001)	.001
Visits by triage category^b^									
Immediate	1359	97	4.8	0.8	4.8 (3.2 to 6.5)	0.8 (0.2 to 1.4)	−4.0	−0.5 (−0.7 to −0.3)	.001
Emergent	3032	1390	9.5	10.1	9.6 (7.4 to 11.8)	8.6 (6.5 to 10.7)	−1.0	−0.3 (−0.5 to 0.01)	.06
Urgent	10 176	5541	32.6	36.8	33.1 (26.2 to 39.8)	31.4 (24.7 to 38.1)	−1.7	−0.4 (−1.5 to 0.8)	.47
Semiurgent	6125	4040	21.6	27.5	21.9 (17.1 to 26.7)	24.6 (18.4 to 30.7)	2.7	0.4 (−0.7 to 1.5)	.43
Nonurgent	3948	680	14.3	45.5	14.5 (10.7 to 18.2)	3.9 (2.5 to 5.2)	−10.6	−0.9 (−1.2 to −0.5)	.001
Mean triage category^c^	NA	NA	NA	NA	3.4 (3.3 to 3.5)	3.3 (3.3 to 3.4)	−0.1	0 (−0.04 to 0.1)	.37

Abbreviations: ACSC, ambulatory care–sensitive conditions; ED, emergency department; NA, not applicable; RD, rate difference; RC, annual rate of change.

^a^ These denominators represent 2008 to 2016 because 2008 was the first year the US Census Bureau provided data in a way to allow for identification of urban and rural populations by payer type.

^b^ Denominator is ED visits for each year; proportions are the percentage of total estimated ED visits. Due to *International Statistical Classification of Diseases and Related Health Problems, Tenth Revision *transition occurring in 2015, we reported ACSCs for 2005 to 2015 to avoid invalid comparisons with 2016 data.

^c^ Calculated by assigning a value of 1 (most acute) through 5 (least acute) to each of the 5 triage categories and then taking the mean for each year with confidence intervals; *P* value is trend during study for mean triage category.

### Demographic Characteristic and Payer Trends

Across urban and rural EDs during the study, each age group demonstrated increase in use, with a more rapid change in rural visits. For rural EDs, 2 groups experienced statistically significant increases: those aged 18 to 44 years (46.9 to 81.6 visits per 100 persons; RD, 34.7; RC, 2.3; 95% CI, 1.1-3.5) and aged 45 to 64 years (27.5 to 53.9 visits per 100 persons; RD, 26.5; RC, 1.6; 95% CI, 0.7-2.5) (Table 1). In contrast, urban EDs experienced increases in the same age groups but at a slower rate. For those aged 18 to 44 years, visits increased from 41.3 to 45.7 visits per 100 persons (RD, 4.4; RC, 0.5; 95% CI, 0.1-0.9); for those aged 45 to 65 years, visits increased from 29.4 to 39.5 visits per 100 persons (RD, 10.1; RC, 0.7; 95% CI, 0.4-1.0) (Table 2).

Among race/ethnicity groups, rural Non-Hispanic white patients demonstrated the largest increases in ED visits (RD, 26.1; RC, 1.6; 95% CI, 0.4 to 2.8). The most notable differences in payer type between rural and urban ED use are for the Medicaid population and patients without insurance. Rural Medicaid visits experienced the largest change and the steepest rate increase from 56.2 to 112.6 per 100 persons (RD, 56.4; RC, 4.1; 95% CI, 2.1 to 6.1), which is in contrast to the urban population’s slower increase, from 56.6 to 88.3 visits per 100 persons (RD, 31.7; RC, 2.9; 95% CI, 1.6 to 4.4). In addition, rural visits by patients without insurance increased significantly during the period studied from 44.0 to 66.6 per 100 persons (RD, 22.6; RC, 2.7; 95% CI, 0.2 to 5.2) in comparison with a small, nonsignificant decrease in urban EDs from 45.7 to 38.8 visits per 100 persons (RD, −6.9; RC, −0.3; 95% CI, −1.6 to 1.0).

### ED Visit Characteristics Trends

Urban and rural EDs experienced small, nonsignificant changes in ambulatory care–sensitive conditions; rural visits increased from 3.6 to 4.5 visits per 100 persons (RD, 0.9; RC, 0.1; 95% CI, 0 to 0.2) in comparison with minimal change in urban proportion of visits. The proportion of rural ED visits that led to hospital admission decreased from 9.3% (95% CI, 4.1% to 14.5%) to 6.3% (95% CI, 1.5% to 11.1%) (RD, −3.0; RC, −0.3; 95% CI, −0.5 to −0.1); the increase in transfer rates, from 3.3% (95% CI, 1.6% to 5.1%) to 4.2% (95% CI, 1.3% to 7.0%) (RD, 0.9; RC, 0.1; 95% CI, 0 to 0.2), was not significant. The overall acuity of ED visits as measured by NHAMCS triage categories lessened in rural EDs over time from a mean value of 3.1 (95% CI, 2.8 to 3.4) in 2005 to 3.4 (95% CI, 3.3 to 3.6; RD, 0.3) (RC, 0.5; 95% CI, 0.2 to 0.7) in 2016, whereas in urban EDs this was largely unchanged over time.

**Figure 2.**
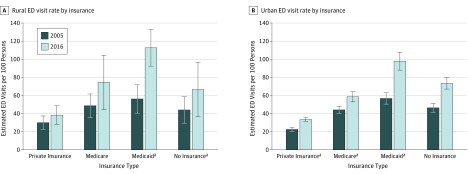
Rural and Urban Emergency Department (ED) Visit Rates by Insurance Type in 2005 and 2016 Proportion of ED visits by insurance type is reported for 2005 and 2016. Error bars represent 95% CIs. ^a^Statistically significant change in the trend of ED visits for all years between 2005 and 2016 (*P* < .05).

In 2005, the estimated count of rural safety-net EDs was 769 of 2009 US rural hospitals (38.3%). By 2016, the number of rural safety-net EDs had increased to 1187 of 1855 rural hospitals (65.0%). A rise in Medicaid visits, which increased proportionally from 25.9% in 2005 to 32.2% in 2016, was associated with this change. During the same period, rural EDs experienced a modest decrease in the proportion of patients without insurance, from 16.3% to 11.1% (Figure 2). In comparison, urban EDs experienced a larger increase in their Medicaid share, from 24.2% in 2005 to 39.9% in 2016, which was offset by a larger decrease in patients without insurance, from 18.5% to 10.1%.

## Discussion

Patterns of ED use provide insight into a community’s health and local care delivery system, thereby serving as potential markers for access and health status. Our study demonstrates that rural EDs have experienced a substantial increase in patient visits from 2005 to 2016—growth of more than 50%—despite a 5% decline in the overall US rural population.^16^ By 2016, nearly one-fifth of all ED visits occurred in the rural setting. Further, while the ratio of rural to urban ED visits was 1:1.1 visits per 100 persons in 2005, a reversal occurred by 2016, when there were 1.5 rural ED visits for every 1 urban ED visit. These changes seem associated in particular with increases in ED use by those aged 18 to 64 years, non-Hispanic white patients, Medicaid beneficiaries, and patients without insurance. Accompanying these changes in demographic characteristics, we also found an increase in lower-acuity rural visits. Finally, the proportion of rural EDs classified as safety-net EDs increased by 26.7% between 2005 and 2016, representing an increased reliance on Medicaid reimbursement.

The disproportionate rise in rural ED visits, particularly for traditionally disadvantaged populations, suggests several considerations for the health of rural residents and rural health care delivery. Increased visits by young to middle-aged white rural patients—particularly Medicaid beneficiaries and those without insurance—may indicate an increased burden of illness or challenges in access to alternative care sites. This has implications for health outcomes, as a greater and increasing reliance on EDs for care by rural patients may complicate efforts to bolster chronic disease management and lead to fragmentation of care. The traditional ED mission focuses on care for acute conditions and therefore may not have resources devoted to modifying health behaviors or addressing long-term conditions. Efforts may be further challenged by additional obstacles unique to rural settings, including fewer personal economic resources,^22^ increased social and geographic isolation, older age, and greater burden of health risk factors, such as obesity,^23^ smoking,^24^ and opioid overdose.^25,26^ In contrast, we found stable ED use rates for the youngest (aged ≤18 years) and oldest (aged ≥65 years) age groups regardless of urban/rural designation. This may reflect better access to primary care and long-term disease management efforts tied to more stable insurance coverage owing to options such as universally available Medicaid for children and Medicare for older adults.

Increases in lower-acuity visits to rural EDs and a similar trend for ambulatory care–sensitive conditions indicate that rural patients may face barriers to timely outpatient ambulatory and primary care services.^27,28^ Rural EDs may be serving as the most immediately accessible source of health care for rural communities. This finding is consistent with the documented intractable rural primary care shortage,^5^ misdistribution of primary care favoring urban centers,^29^ and rapid rural primary care physician turnover,^30^ all of which may contribute to increased ED use. Previous studies suggest that poor primary care access is associated with increased ED use,^31,32^ with rural patients less likely to have a primary care follow-up visit and more likely to have an ED visit following an inpatient admission.^33^ Historically, the use of EDs for routine and primary care conditions is perceived as low value, with efforts to reduce ED use in urban communities and health systems focused on investments in care coordination^34,35,36^ and medical homes.^37,38^ Recent attention to the decline in rural health has prompted calls for rural hospitals and clinicians to more forcefully embrace these population health management principles.^39^ However, these approaches require significant practice transformation, adequate resource investment, economies of scale, and a robust physician pool—all which may be lacking in the rural setting.

Therefore, rural areas may require tailored and innovative strategies to achieve improvements in the access to and availability of health care in their communities. While existing federal support for these programs will continue, the traditional approaches to bolstering primary care, including the National Health Service Corps program, foreign medical graduates, and primary care residency training in rural communities, have yielded mixed returns while facing variable financial support.^40,41,42^ Telehealth is another promising strategy, but it has struggled with large-scale implementation in rural areas because of significant development costs and reimbursement challenges, despite significant interest by primary care physicians.^43^ Innovation in acute care delivery occurring in the urban setting, including urgent care clinics, home monitoring, and e-visits, have had poor penetration into rural health care delivery. This is in part owing to a lack of patient volume to support such innovations as well as limitations in telecommunication infrastructure. It has been increasingly recognized that rural-specific innovations are needed, yielding the concept of emergency medical centers (also known as *rural freestanding EDs*), in which existing hospitals transition to a facility divested of inpatient beds.^44^ These facilities then focus care on targeted outpatient services in coordination with an on-site comprehensive ED.^45^ Alternatively, for smaller communities, a primary care clinic with extended hours could be linked with an ambulance service operating 24 hours a day, every day. Through these strategies advanced by the Centers for Medicare & Medicaid Services,^46^ rural hospitals can focus on outpatient management of long-term conditions and high-risk health behaviors while simultaneously ensuring high-quality treatment for acute conditions and rapid transfer of patients requiring hospitalization to larger centers.

We additionally found an increase in the proportion of rural EDs classified as safety-net EDs, indicating an erosion in rural hospitals’ payer mix. Underlying this trend is an increasing reliance on Medicaid for reimbursement without a commensurate decline in visits by patients without insurance, which contrasts with the urban ED experience. These trends may be associated with Medicaid expansion in states with large rural populations, as Medicaid expansion in such states increased coverage for low-income rural adults.^47^ Even with Medicaid expansion, rural hospitals operate on thin margins, often requiring special payment programs to remain financially viable.^48^ Despite these federal efforts, more than 90 rural hospitals have closed in the last 10 years,^49^ threatening rural communities’ access to necessary local health care. These developments are partially the result of reductions in inpatient admissions nationwide as well as market trends promoting increasing hospital and health system consolidation.^50^ Further, states with large rural populations have generally been reluctant to expand Medicaid, which may be related to most rural hospital closures occurring in those states.^51^ This may be reflected in the proportion of visits to rural EDs by patients without insurance, which experienced only a 5% decrease compared with a 9% decrease that occurred in urban EDs. In response to these cumulative financial pressures on rural hospitals, some states are experimenting with an alternative payment model of global rural health care budgets. Maryland’s rural hospitals are paid a fixed amount in advance for inpatient and outpatient hospital-based services. Pennsylvania is now attempting the same^52^ with hopes that the greater certainty of prospective funding should allow rural hospitals to better invest in necessary quality and preventive care.^53^ Our findings suggest that increased Medicaid reimbursement would help stabilize rural hospitals in a traditional fee-for-service model and alternative payment models, like global budgets, may be a more successful strategy given the deteriorating payer mix noted at rural EDs.

### Limitations

There are several limitations of this study related to the NHAMCS survey design and assumptions tied to some of the study’s outcome measures.^54^ First, NHAMCS does not provide unique patient identifiers; therefore, the extent to which these visits represent repeated visits for the same patients or new patients is unknown. Second, methods for determining ambulatory care–sensitive conditions were designed for hospital inpatient discharge data, but applying them to ED discharge diagnoses has been described successfully.^55^ Third, there are more than 15 ways to define rurality, which complicates urban vs rural analyses. The NHAMCS categorizes hospitals into urban or rural in alignment with the Office of Management and Budget, which relies on Metropolitan Statistical Areas. To match the NHAMCS convention, we defined our urban and rural reference populations by the US Census Bureau according to these same criteria. Fourth, while it is important to recognize that rural areas are heterogeneous and the findings reported may vary from one type of rural location to another, we were unable to explore more granular geographic estimates with this data set. For example, as there are no state identifiers in NHAMCS, we were unable to determine how the ED payer mix changed in Medicaid expansion vs nonexpansion states. In addition, NHAMCS allows parsing at the regional level, but our analysis in these cases was subject to a sample size less than 30, which produces unstable estimates.^54^ Fifth, this is national survey data, and our findings are hypothesis generating; these findings will need to be explored in other data sets for confirmation of these trends. However, despite these limitations, the national trends reported in this analysis remain an important insight into the overall experience in rural health care delivery.

## Conclusions

These findings demonstrate several important and concerning implications for rural population health care delivery. Increased ED use may reflect a deteriorating primary care infrastructure, greater fragmentation of care, and worsening disparities for several traditionally disadvantaged groups, including those with Medicaid and those without insurance. Additionally, rural EDs are increasingly serving as safety-net hospitals, potentially further destabilizing their budgets because they generally operate in the traditional fee-for-service model. To improve the health of individuals in the rural United States, improved Medicaid reimbursement and innovative payment and delivery models that integrate EDs into local health care delivery systems may prove successful.

## Notes

Supplement. **eTable.** Detailed statistical appendix regarding visit count, rates, standard error, and weighting
