# Supplementary material for: Trends in Emergency Department Use by Rural and Urban Populations in the United States
Source: JAMA Netw Open. 2019 Apr 12;2(4):e191919. doi: 10.1001/jamanetworkopen.2019.1919 (PMC6481434; doi:10.1001/jamanetworkopen.2019.1919)
Supplement: Supplement. — eTable. Detailed statistical appendix regarding visit count, rates, standard error, and weighting [file jamanetwopen-2-e191919-s001.pdf]

Supplementary Online Content

Greenwood-Ericksen MB, Kocher K. Trends in emergency department use by rural and urban populations in the United States. *JAMA Netw Open.* 2019;2(4):e191919. doi:10.1001/jamanetworkopen.2019.1919

eTable. Detailed statistical appendix regarding visit count, rates, standard error, and weighting

This supplementary material has been provided by the authors to give readers additional information about their work.

© 2019 Greenwood-Ericksen MB et al. *JAMA Network Open.*

eTable 1. Detailed statistical appendix regarding visit count, rates, standard error, and weighting

URBAN					
**Year**	**Visit Count (95% CI)**	**US Urban (MSA) Population***	**Rate (95% CI)**	**Standard Error**	**Weight = 1/variance**
2005	98,622,469 (81,234,682, 116,010,256)	245,444,440	40.18 (33.10, 47.27)	8,863,305	0.0766858
2006	100,727,687 (82,274,915, 119,180,459	244,770,063	41.15 (33.61, 48.69)	9,406,174	0.0677159
2007	99,074,312 (78,370,636, 119,777,988)	249,078,372	39.78 (31.46, 48.09)	10,553,557	0.05570244
2008	103,820,096 (84,266,638, 123,373,554)	250,112,038	41.51 (33.69, 49.33)	9,967,241	0.06296792
2009	111,461,968 (88,821,565, 134,102,371)	254,253,975	43.84 (34.93, 52.74)	11,540,790	0.04853606
2010	107,799,057 (88,480,562, 127,117,552)	256,865,185	41.97 (34.45, 49.49)	9,847,470	0.06803949
2011	114,888,677 (94,128,378, 135,648,976)	257,417,133	44.63 (36.57, 52.70)	10,582,420	0.05917044
2013	104,776,211 (80,897,140, 128,655,282)	268,235,178	39.06 (30.16, 47.96)	12,172,193	0.04856169
2014	118,182,880 (91,297,516, 145,068,244)	272,914,209	43.30 (33.45, 53.16)	13,704,630	0.0396568
2015	118,059,100 (94,409,200, 141,709,000)	276,538,559	42.70 (34.14, 41.24)	12,056,774	0.05260768
2016	117,189,336 (93,067,901, 141,310,772)	273,981,761	42.78 (33.97, 51.58)	12,297,164	0.04964018

RURAL

Year	Visit Count (95% CI)	US Rural (Non-MSA) Population*	Rate (95% CI)	Standard Error	Weight = 1/variance
2005	16,700,346 (7,842,509, 25,558,183)	45,711,479	36.53 (17.16, 55.91)	4,515,222	0.01024926
2006	18,463,841 (8,464,935, 28,462,747)	49,410,091	37.37 (17.13, 57.61)	5,096,874	0.00939774
2007	17,727,754 (8,586,561, 26,868,947)	47,224,036	37.54 (18.18, 56.90)	4,659,661	0.01027112
2008	19,941,323 (9,693,470, 30,189,176)	48,576,121	41.05 (19.96, 62.15)	5,223,773	0.00864723
2009	24,610,162 (10,600,347, 386,199,77)	47,316,367	52.01 (22.40, 81.62)	7,141,407	0.00438991
2010	22,044,320 (9,208,374, 34,880,266)	46,762,696	47.14 (19.69, 74.59)	6,543,035	0.00510788
2011	21,407,723 (9,103,970, 33,711,476)	48,961,619	43.72 (18.59, 68.85)	6,271,753	0.00609444
2013	25,576,870 (13,184,553, 37,969,187)	42,694,248	59.91 (30.88, 88.93)	6,316,899	0.00456805
2014	23,237,580 (10,914,081, 35,561,079)	40,758,651	57.01 (26.78, 87.25)	6,281,819	0.00420987
2015	18,884,081 (8,789,368, 28,978,794)	39,747,693	47.51 (21.65, 73.37)	5,146,308	0.0059653
2016	28,401,872 (13,199,364, 43,604,380)	44,033,556	64.5 (29.3, 99.7)	7,750,274	0.003228

* Source: US Census Bureau's American Community Survey and Centers for Disease Control's National Center for Health Statistics

© 2019 Greenwood-Ericksen MB et al. *JAMA Network Open.*
